# Vernalization treatment induces site-specific DNA hypermethylation at the VERNALIZATION-A1 (VRN-A1) locus in hexaploid winter wheat

**DOI:** 10.1186/1471-2229-13-209

**Published:** 2013-12-11

**Authors:** Abdul Rehman Khan, Jérôme Enjalbert, Anne-Charlotte Marsollier, Agnès Rousselet, Isabelle Goldringer, Clémentine Vitte

**Affiliations:** 1 INRA, UMR de Génétique Végétale, Gif sur Yvette F-91190, France; 2 CNRS, UMR de Génétique Végétale, Gif sur Yvette F-91190, France

**Keywords:** *VRN1*, Vernalization, DNA methylation, Non CG methylation, Winter wheat, Transposable element, *Triticum aestivum*, Cold, Intron, *Jorge*, *Sumaya*

## Abstract

**Background:**

Certain temperate species require prolonged exposure to low temperature to initiate transition from vegetative growth to flowering, a process known as vernalization. In wheat, winter cultivars require vernalization to initiate flowering, making vernalization requirement a trait of key importance in wheat agronomy. The genetic bases of vernalization response have been largely studied in wheat, leading to the characterization of a regulation pathway that involves the key gene *VERNALIZATION1 (VRN1)*. While previous studies in wheat and barley have revealed the functional role of histone modification in setting *VRN1* expression, other mechanisms might also be involved. Here, we were interested in determining whether the cold-induced expression of the wheat *VRN-A1* gene is associated with a change in DNA methylation.

**Results:**

We provide the first DNA methylation analysis of the *VRN-A1* gene, and describe the existence of methylation at CG but also at non CG sites. While CG sites show a bell-shape profile typical of gene-body methylation, non CG methylation is restricted to the large (8.5 kb) intron 1, in a region harboring fragments of transposable elements (TEs). Interestingly, cold induces a site-specific hypermethylation at these non CG sites. This increase in DNA methylation is transmitted through mitosis, and is reset to its original level after sexual reproduction.

**Conclusions:**

These results demonstrate that *VRN-A1* has a particular DNA methylation pattern, exhibiting rapid shift within the life cycle of a winter wheat plant following exposure to particular environmental conditions. The finding that this shift occurs at non CG sites in a TE-rich region opens interesting questions onto the possible consequences of this type of methylation in gene expression.

## Background

Plants use environmental signals to modify and adapt their growth and development according to local climatic/ecological conditions. A major step in plant life cycle is the transition from the vegetative to the reproductive stage that controls flowering, a critical trait for plant fitness. Seasonal cues, such as temperature and day-length, ensure that flowering coincides with favorable conditions to escape stress and maximize photosynthesis and seed production [1]. In temperate climate, certain species, including the temperate cereals (such as wheat and barley) and dicot species (such as *Arabidopsis* and sugar beet) need to be exposed to a period of prolonged low winter temperatures to accelerate the progression from vegetative to reproductive growth, a process known as vernalization [2-6]. Vernalization requirement is an adaptive trait that prevents flowering initiation prior to winter, which would otherwise result in severe frost damages on fragile flower meristems. The prolonged cold period is remembered over time, maintaining flowering stimulation when temperatures rise back during spring [2,4,7]. The vernalization signal is transmissible through mitosis but is reset in the next sexual generation therefore ensuring that descendants will be themselves able to respond to vernalization.

In *Arabidopsis*, cold induces a transcriptional repression of the repressor of floral initiation *FLOWERING LOCUS C* (*FLC*). This repression is mediated by an epigenetic-based regulation in which the *FLC* chromatin state is switched from an actively transcribed state (with high levels of histone H3 acetylation and histone H3 lysine 4 di- and trimethylation) to a repressed state (with high levels of histone H3 lysine 9 dimethylation, histone H4 arginine 3 symmetrical dimethylation, and histone H3 lysine 27 di- and trimethylation) [3,8,9]. This transcriptional repression is then maintained over cell divisions by mitotic inheritance of the repressive histone modifications [10], but is reset during reproduction, thus allowing progeny to be competent to respond to vernalization [11]. Molecular basis of such transient memory of a cold period during plant development has been recently described in other species. In temperate cereals such as the triticeae, response to vernalization is mediated by the stable induction of a floral activator, *VERNALIZATION1* (*VRN1*) [4,12-14]. *VRN1* encodes a FRUITFULL-like MADS-box transcription factor that is required for the initiation of reproductive development at the shoot apex [15-17]. *VRN1* is central in the vernalization pathway [18] as it down regulates the floral repressor *VERNALIZATION2* (*VRN2*) but also interacts with other flowering pathways, allowing long-day induction of the floral activator *FLOWERING LOCUS T-like 1* (*FT1*) to accelerate subsequent stages of floral development [4,19-22]. The expression of *VRN1* is induced by cold treatment, is maintained when cold treatment is released, and is reset in the next generation [12-14,19,23,24], some characteristics that point out to a possible epigenetic regulation.

The epigenetic regulation of *HvVRN1*, the *VRN1* gene of barley (*Hordeum vulgare*), has been assessed through the analysis of histone post-translational modifications [25]. Vernalization increases active histone marks for transcription (H3K4me3, histone 3 lysin 4 trimethylation) in two regions located in exon one and the beginning of intron 1, while decreasing silent marks (H3K27me3, histone 3 lysine 27 trimethylation) in 6 regions located from the promoter to the end of intron 1. In a more recent study [26], histone acetylation of intron 1 was also shown to be involved in the regulation of the gene: acetylation levels of histones 3 and 4 increase during cold treatment in intron 1, and sodium butyrate, a histone deacetylation inhibitor, induces an increase in *HvVRN1* expression. Altogether, this suggests that in barley vernalization induces histone modifications associated with an active chromatin state, which correlates with an increase in *VRN1* transcripts. These changes are retained posterior to vernalization, providing a molecular hypothesis for the epigenetic-based memory of vernalization in barley.

In hexaploid wheat (*Triticum aestivum*), analysis of H3K4me3 and H3K27me3 histone modifications of the *TaVRN1* promoter region (from two subregions located near the ATG and 1 kb upstream of it) in winter and spring wheat revealed no significant changes for H3K27me3 following vernalization in both genotypes [27]. However, vernalization caused an enrichment of H3K4me3 in winter wheat while a decrease of this histone modification was observed in spring wheat. Altogether, these results suggest that vernalization promotes an active state of the *TaVRN1* chromatin in winter wheat and a reduction of this active state in spring wheat. These results are consistent with the relative abundance of *TaVRN1* mRNA in winter and spring wheat and suggest that the wheat vernalization responsive gene *VRN1* is regulated, at least in part, by histone methylation at the promoter. Genetic studies have shown that, besides the promoter region, internal regions of *VRN-A1* such as intron 1 are also involved in the diffe rential regulation observed between spring and winter wheat [28]. This, together with the results obtained for barley, suggests that an epigenetic regulation of the internal regions of the gene could be involved in the regulation of *VRN-A1*.

However, these studies of the *VRN1* regulation were limited to the analysis of histone marks, and did not allow for testing the potential role of DNA methylation in the cereal vernalization process. While DNA methylation could not explain the expression changes observed for *FLC* in *Arabidopsis*[29], studies on sugar beet (*Beta vulgaris*) revealed that the flowering repressor *BvFLC* is hypermethylated and decreases during vernalization in genotypes sensitive to vernalization, this effect being more pronounced for longer duration of cold treatment [5]. The differences observed between *Arabidopsis* and sugar beet suggest that the ability to respond to vernalization may have been acquired through different molecular mechanisms within dicotyledonous plants and highlight the need to better characterize the role of DNA methylation in the molecular mechanisms involved in the response to vernalization for other species. In particular, the role played by DNA methylation in the vernalization response of monocotyledonous plants such as cereals needs to be investigated.

DNA methylation on cytosines is a well studied epigenetic mark in plants. It is involved in the regulation and transcriptional silencing of genes and transposable elements (TEs), respectively, and plays major roles in cellular differentiation and genome evolution [30,31]. Inherited methylation changes also likely play a role in plant phenotypic evolution [32,33]. While DNA methylation mainly occurs at CG sites in animal genomes, it can affect cytosines in CG, CHG and CHH contexts (H denotes A, C or T) in plants [34,35]. Repeated sequences such as TEs are heavily methylated at the three cytosine contexts, whereas DNA methylation in the body of the genes (gene body methylation) is less dense and mainly limited to CG sites [20,35-38]. Whereas the mechanisms underlying the establishment and maintenance of DNA methylation are now relatively well understood, little is known about their dynamics during the plant and animal life time. Several studies have focused on the biological relevance of DNA methylation dynamics in plant response to biotic stresses [39,40] but its relevance in the response to abiotic cues remains largely unknown.

In this article, we investigated the effect of vernalization on the DNA methylation profile of the *VRN-A1* gene in winter wheat. We focused on studying the cellular memory of cold-induced DNA methylation after the environmental stimulus was removed. For this, we analyzed the DNA methylation pattern of *VRN-A1* on plants submitted or not to 45 days-long cold treatment inducing the expression of *VRN-A1* gene and placed back in greenhouse conditions for 10 additional days.

Our study indicates that cold induces an increase in DNA methylation within the body of the *VRN-A1* gene, which is reset in the next generation, thus highlighting a first case of environmentally-induced epigenetic change in wheat. Whether the observed increase in DNA methylation explains the increase in expression of the *VRN-A1* gene or is simply correlative remains to be elucidated. Nevertheless, the finding that cold-induced hypermethylation specifically affects non CG sites located in TE fragments from the 8.5 kb long intron 1 suggests that TE fragments may facilitate the DNA hypermethylation process observed.

## Results

### Overall DNA methylation pattern of VRN-A1 in non-vernalized plants

**Figure 1:**
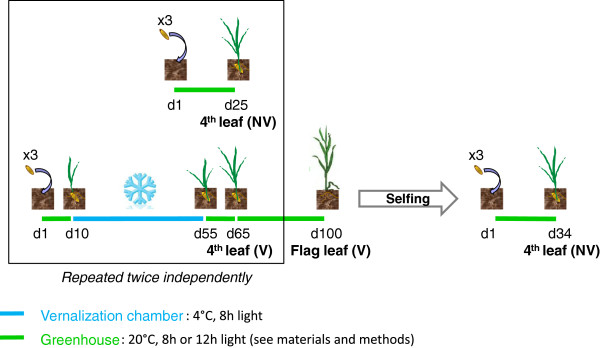
**Experimental design highlighting vernalization procedure.** The experimental design was made in two steps (i) to investigate differences in DNA methylation between vernalized and non vernalized plants at the 4^th^ leaf stage, and (ii) to investigate the mitotic and transgenerational inheritance of the DNA methylation signal. In these two sets of experiment, comparison of the *VRN-A1* DNA methylation profile was performed for vernalized and non vernalized plants at the 4^th^ leaf stage, thus allowing to check for repeatability of the signal (two independent sets of experiment). For each experiment and each stage (4^th^ leaf, flag leaf, progeny 4^th^ leaf), 3 independent plants were analyzed, as shown by the “x3” sign. Stages and type of leaf sampled are shown at the bottom each plant analyzed, in bold. Growing conditions are represented by blue (vernalization chamber) and green (greenhouse) lines, with length corresponding to the number of days on which the treatment was applied. Abbreviations: NV: non vernalized, V: vernalized, d: day.

DNA methylation pattern of the *VRN-A1* gene was investigated using the bisulfite conversion method, which allows for the quantitative detection of methylation level for each cytosine of a PCR-amplified fragment. To analyze the overall methylation pattern of the *VRN-A1* gene, we analyzed 9 fragments located along the *VRN-A1* gene, covering 21% of this 12 kb gene (Table 1). DNA methylation of these 9 fragments was investigated on two genotypes, three biological repetitions and two treatments (with and without 45 days exposure at 4°C, Figure 1). Because these experiments would be facilitated by the use of a cost- and labor-effective method to quantify DNA methylation, we used direct reading of PCR Sanger sequence chromatograms to quantify DNA methylation at each cytosine site after bisulfite conversion and PCR amplification (see Additional file 1 for details). Design of primers fitting the bisulfite treatment requirements for a hexaploid genome was not trivial, and about 50% of the PCR designed were either not amplifying or leading to poor sequencing traces. In particular, the middle of intron 1 and the region located between exons 4 and 8 could not be covered by bisulfite-PCR assays. We therefore used methylsensitive PCR assays (5 fragments) to investigate the DNA methylation profile of these regions (Table 2 for enzyme specificity and 3 for PCR amplicons). We also used this technique to validate results for 3 bisulfite-PCR fragments with contrasted DNA methylation patterns (Table 3, and Additional file 1 for comparison of the two patterns).

**Table 1:** Characteristics of PCR amplicons used for bisulfite analysis

**Fragment number**	**Primer name**	**Primer sequence (5′-3′)**	**Position of fragment within the** ***VRN-A1*** **gene (bp from start of Genbank accession AY747600)**	**Size of amplified fragment (bp)**
0.01 k^(1)^	0.01 k-F	AAATGATTTGGGGAAAG**T**AAAAT**T**	100–311	212
	0.01 k-R	**A**A**AAAA**TTTTTAAA**AAA**ATCT**AA**CCC		
1.2 k^(1)^	1.2 k-F	AT**T**AAAT**T**TGTG**T**TTG**T**TG**T**TTGA	1221–1468	248
	1.2 k-R	AACTCTCTACTTTTT**AA**TTT**A**ACTCTTC		
2.2 k	2.2 k-F	GGGGA**T**AAGTAA**T**TG**T**TATG**T**TTTG	2259–2490	233
	2.2 k-R	**A**TCCA**A**AAAATCA**A**CA**AA**CTACAT		
6.8 k(a) ^(2) (3)^	6.8 k(a)-F	TTG**T**TTG**T**ATGTGAG**T**AGA**T**TGGA	6847–7147	301
	6.8 k(a)-R	**AA**CTACTCCTCCACCTTAT**A**CCAA		
7.6 k^(4)^	7.6 k-F	TGAGGAGG**T**TGGAAG**T**ATTAAG**T**A	7679–7393	315
	7.6 k-R	T**A**CACCCCT**A**CA**AAA**CTAAATCTT		
9.2 k	9.2 k-F	ATG**T**AGTATGGA**T**AAAATT**T**TTGAA	9203–9711	509
	9.2 k-R	TT**A**TT**A**CTAA**A**CCCTTCAAAAACTC		
10.1 k	10.1 k-F	GTTGTAG**T**T**TT**AA**T**TGAGGGAT**T**	10130–10512	383
	10.1 k-R	ACCCCT**A**TCCATA**A**CTA**A**CTCT		
10.5 k	10.5 k-F	AA**T**TTGTTTGGGA**T**TAAAGG**T**T	10592–10939	340
	10.5 k-R	CAA**A**ATCCTCTCCCAT**A**A**A**AT**A**C		
11.7 k	11.7 k-F	GTGTT**Y**G**T**TTTGGTTGTG**T**AG**T**	11792–12048	257
	11.7 k-R	ACTCT**A**ATTTCTTTTCCTTTCCC		

Bold letters in the sequence represent primer positions matching cytosines, which were considered as non methylated and therefore converted into thymines after bisulfite treatment (Cs were changed to Ts in forward primers and Gs to As in reverse primers). ^(1)^Primer pairs that were not genome A specific; ^(2)^Fragment for which several primer pairs with different C/T content were designed (see Additional file 1); ^(3)^While corresponding PCR amplicon contains a fragment of the *Jorge* transposon, the reverse primer does not overlap the annotation of this element. The closest Jorge element found shares only 76% nucleotide identity with this amplicon; ^(4)^While the two primers are included within the *Sumaya* annotation, the closest *Sumaya* element found shares only 81% nucleotide identity with this amplicon.

**Table 2:** Recognition sites and methylation sensitivities of the restriction enzymes used

**Enzyme**	**Recognition site**	**Sensitivity to methylation**
*Bfa* I		none
*BstU* I		CG
*Hpa* II		CG
*Msp* I		CHG
PspG I		CHG/CHH

Arrowheads indicate enzymes cutting sites and bold Cs show methylation sensitive sites.

**Table 3:** Description of amplicons characteristics used for restriction enzyme analyses

**Fragment number**	**Primer name**	**Sequence (5′-3′)**	**Position of fragment within the** ***VRN-A1*** **gene (bp from start of Genbank accession AY747600)**	**Size of amplified fragment (bp)**	**Number of restriction site within amplicon for:**		
					***Msp*** **I**	**PspGI**	***Bst*** **UI**
**0.0 k**	VRN_1_A_pr_F*	GAAAGGAAAAATTCTGCTCG	44–527	483	8	1 (n.a.)	3
	VRN_1_ex1_R*	TGCACCTTCCCSCGCCCCAT					
**1.6 k**	1.6 k-F	GCCTCCACGGTTTGAAAGTA	1695–2358	663	1	0	2
	1.6 k-R	ATCTCAAGATTTTAGTTCCGATCCT					
**3.0 k**	3.0 k-F	TGCTGCAGTGATATTTTGTTAGC	3030–3710	680	0	1	0
	3.0 k-R	TGATGGGTCATAAGGTTTTGC					
**4.0 k**	4.0 k-F	CTTCCTTGGTGGGCTGTG	4003–4607	604	1	0	1
	4.0 k-R	TGGCTCTCCACCACAATACC					
**5.0 k**	5.0 k-F	GGCTAAGATCGTGAGGAAGG	5014–5648	634	0	1	0
	5.0 k-R	TGACTAGCACCACATCAATCG					
**6.8 k (b)**	6.8 k (b)-F	GCGGCATCATCTTCTTGC	6834–7148	314	1	0	2
	6.8 k (b)-R	GGCTACTCCTCCACCTTATGC					
**9.8 k**	9.8 k-F	TCTCCAGTCCTTCGGATTGT	9871–10489	618	1	1	0
	9.8 k-R	GGCTTTTGGGTTTCATCTCC					
**11.0 k**	11.0 k-F	AATGATTTGATACAGCAGCACAATA	11028–11710	682	1	1	0
	11.0 k-R	ACCAATTCAAAAGATGGTTACTTGA					

*PCR primers from [41]. Abbreviations: n.a.: not analyzed.

**Figure 2:**
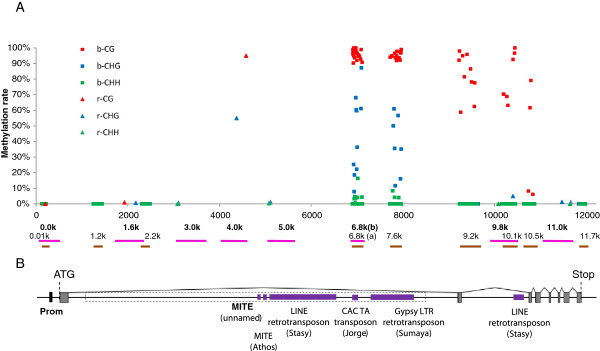
**DNA methylation profile across the 12 kb –long *VRN-A1 *gene. A**. Site by site DNA methylation levels across the *VRN-A1* gene. Squares and triangles represent bisulfite and restriction enzymes data, respectively. Red, blue and green colors represent CG, CHG and CHH contexts, respectively. **B**. Gene structure of the *VRN-A1* gene (based on the Genbak accession AY747600). The black box indicated as “Prom” highlights position of the promoter region as described in [41]. Grey boxes represent exons. Dashed frame indicates position of the deletion in the Langdon allele (from alignment comparison with Genbank accession AY747598). Purple lines highlight positions of transposable element sequences, with corresponding class and name indicated below. Brown and pink lines represents the fragments studied using bisulfite-based and restriction enzyme-based techniques, respectively. Corresponding fragment names are given above each line.

**Figure 3:**
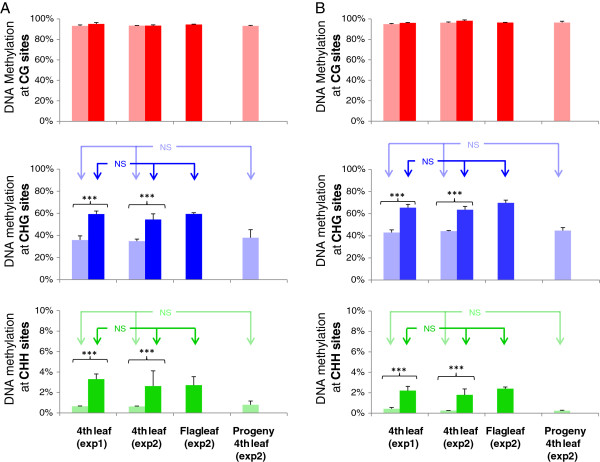
**DNA methylation levels for 4**^**th **^**leaf, flag leaf and progeny 4**^**th **^**leaf. A**. Fragment 6.8 k (a). **B**. Fragment 7.6 k. Results of both G1 and G2 were pooled. Red, blue and green colors represent CG, CHG and CHH contexts, respectively. Faint and strong colors represent non-vernalized and vernalized plants respectively. From left to right: DNA methylation from on 4^th^ leaf for two independent sets of experiments (exp 1 and exp 2), from the flag leaf of exp 2 and for the 4^th^ leaf of the progeny (exp 2). In experiment 1, results from three replicates of G1 and three replicates of G2 were pooled. In experiment 2, results from two replicates of G1 and two replicates of G2 were pooled. Stars highlight statistical significance of methylation variation induced by vernalization treatment, with ***p < 0.001.

Analysis of these 17 fragments (covering 540 cytosines) on one plant from the G1 genotype grown in non vernalizing conditions revealed that most fragments (0.0 k, 0.01 k, 1.2 k, 1.6 k, 2.2 k, 3.0 k and 11.7 k) were not methylated. For the remaining fragments, DNA methylation was either restricted to CG sites (fragments 9.2 k to 10.5 k), or was observed in all CG, CHG and CHH contexts (fragments 4.0 k, 6.8 k(a) and 7.6 k). These results indicate that the DNA methylation of the *VRN-A1* gene is heterogeneous along its sequence, with an absence of methylation at the start and end, while the region extending from the middle of intron 1 to the start of exon 4 is highly methylated (Figure 2). DNA methylation rates at the different cytosine contexts (Figures 2 and 3) were similar to what has been observed in *Arabidopsis* and maize (typically 80-100% for CGs, 20-100% for CHGs and up to 10% for CHHs; [38,42,43].

### Vernalization induces both hypermethylation at the end of VRN-A1 intron 1 and gene transcription increase

Comparison of DNA methylation profiles of one vernalized and one non-vernalized plant of the G1 genotype for all bisulfite PCR fragments revealed a difference of methylation level only for fragments 6.8 k(a) and 7.6 k. Further investigation of the DNA methylation pattern of these two fragments on two additional biological replicates of genotype G1 and three biological replicates of genotype G2 showed that patterns were homogeneous among non-vernalized plants, and among vernalized plants, and confirmed the difference observed between vernalized and non vernalized plants (Figure 3, exp. 1). CG sites show an average methylation rate higher than 90% in both conditions, with no significant variation between treatments. In contrast, CHG and CHH sites showed a significant increase in DNA methylation in vernalized plants (Figure 3, exp.1).

**Figure 4:**
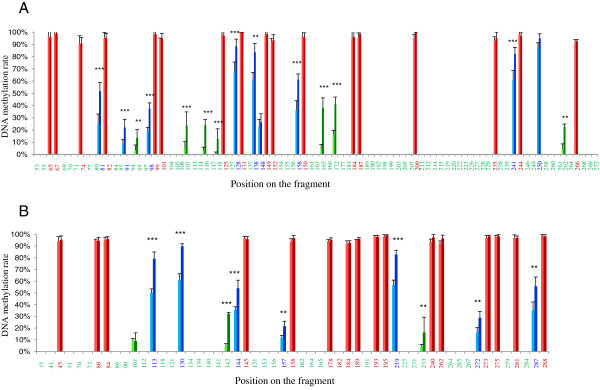
**Site by site DNA methylation variation between vernalized and non-vernalized plants. A**. Fragment 6.8 k (a). **B**. Fragment 7.6 k. Stars highlight statistical significance of methylation variation induced by vernalization treatment, with **p < 0.01 and ***p < 0.001. Red, blue and green colors represent CG, CHG and CHH contexts, respectively. Faint and strong colors represent non-vernalized and vernalized plants respectively.

Analysis of cytosines taken individually (Figure 4 and Additional file 2: Figure S1) revealed that all CG sites are methylated above 90% in both conditions, while all CHG sites are methylated in non vernalized plants but show a significant increase in DNA methylation following vernalization (except sites 148 and 250 of fragment 6.8 k(a) for which the increase is visible but not significant). Interestingly, while only a small fraction of CHH sites is methylated (11.3% and 0.4%, in fragment 6.8 k(a) and 7.6 k, respectively), no additional methylated site was found. Rather, a significant increase of DNA methylation was observed for almost all sites that were methylated in non-vernalized conditions (Figure 4). Comparison of the two genotypes revealed very similar methylation patterns: only sites 118 and 165 of fragment 6.8 k(a) showed significant differences between the two genotypes (G1 showed 4.8 and 1.5 times higher DNA methylation than G2 at sites 118 and 165 respectively; data not shown).

Taken together, these results reveal that, at the end of intron 1, vernalized plant show an increase in methylation at CHG and CHH sites that is restricted to the sites that are also partially methylated in non-vernalized plants. On the contrary, almost all CG sites are highly methylated in both conditions, and are therefore not impacted by the vernalization treatment. These observations were confirmed on a second set of experiments (Figure 3, exp. 2), indicating that the shift in DNA methylation observed is repeatable.

**Figure 5:**
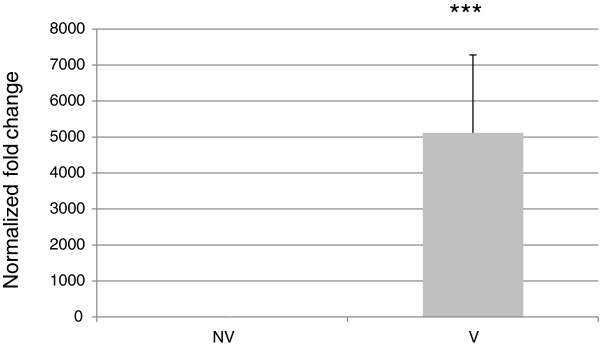
**Relative expression of *VRN-A1 *in vernalized and non vernalized plants (4**^**th **^**leaf).** Data represent the mean ± standard deviation from 2 biological of the G1 and G2 genotypes. Two technical replicates gave the same results. The level of expression is normalized by this obtained for *18S.* For comparison, results are calibrated by the non-vernalized point.

Because the expression of the *VRN-A1* gene is known to increase following vernalization [23], our data suggested a positive correlation between DNA methylation and gene transcription. To identify whether the DNA methylation shift observed following vernalization was correlated to the expression of the *VNR-A1* gene, we performed qRT-PCR for both vernalized and non-vernalized conditions, using the same leaf powder used for DNA methylation analyses. qPCR results indicate a 10 Ct change between vernalized and non vernalized plants, pointing to a 1000 fold increase of *VRN-A1* leaf mRNA levels following vernalization (Figure 5). This expression increase is associated with the DNA methylation shift observed, with *VRN-A1* being expressed when the gene shows higher gene body methylation.

### The high level of DNA methylation observed in vernalized plants is maintained through mitosis but is reset in the next generation

To verify the results obtained on 4^th^ leaf and to study the mitotic and transgenerational transmission of the modifications in DNA methylation observed following vernalization, a second set of plants was grown up to flowering maturity. DNA methylation pattern of fragments 6.8 k(a) and 7.6 k was analyzed on fourth leaf and flag leaf samples (Figures 1 and 3). No significant difference was observed between the 4^th^ leaf and the flag leaf samples (Figure 3), thus revealing that the DNA methylation profile observed is maintained through mitosis.

Vernalized plants of this second set were selfed, and the progeny was analyzed in non-vernalizing conditions at the fourth leaf stage (Figure 1). DNA methylation profile analysis of fragments 6.8 k(a) and 7.6 k revealed that all cytosines show a methylation profile typical of non vernalized plants (Figure 3), therefore providing evidence that the increase in DNA methylation induced by vernalization is reset in the next generation.

### The sequence of winter allele of VRN-A1 harbor TE fragments not found in VRN-B1 and VRN-D1 copies

**Figure 6:**
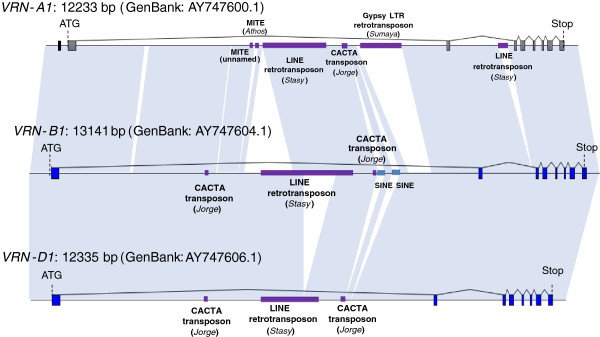
**Comparative analysis of the *VRN-A1*, *VRN-B1 *and *VRN-D1 *copies highlighting different TE content.** Accession numbers used to represent the three genes are shown on the left, with corresponding size. The three genes are drawn at the same scale. Grey *(VRN-A1*) and dark blue (*VRN-B1* and *VRN-D1*) rectangles represent exons. The black box in *VRN-A1* highlights position of the promoter region as described in [41]. Purple lines highlight positions of transposable element sequences as defined by our annotation, with corresponding class and name shown below. Blue lines represent the annotation of two SINE elements that could not be found in our analysis but were described in GeneBank accession AY747604.1 [28]. Light blue parallelograms represent conserved regions as defined by our GEvo analysis.

TE annotation within the *VRN-A1* winter genotype sequence using the most recently updated wheat transposable element database [44] revealed the presence of several TE fragments, most of which are located within the second half of intron 1 (Figure 2). Interestingly, the region covered by these TEs is the region showing the highest methylation level, harboring methylated Cs from CG context, but also from CHG and CHH contexts. In particular, fragments 6.8 k(a) and 7.6 k that show a difference in DNA methylation level between vernalized and non-vernalized plants also harbor pieces of TEs: amplicon 6.8(a) contains a fragment of the CACTA-like TIR transposon *Jorge* (126 bp of the internal part of the element, from base 6854 to base 6979) while amplicon 7.6 k is fully covered by a piece of the *gypsy*-like LTR retrotransposon *Sumaya* (952 bases of the LTR, from position 7213 to 8164). Annotation of *VRN-B1* and *VRN-D1* genes and comparative analysis with *VRN-A1* showed that the *Sumaya* element is specific from the A genome *VRN1* copy (Figure 6). Comparison of the Jorge and Sumaya fragments located in *VRN-A1* with sequences from other Jorge and Sumaya elements extracted from chromosome 3B revealed that they share 76% and 81% identity with the closest copy, respectively. This, together with the fact that they are truncated TEs, suggests that these sequences have evolved from ancient insertions.

## Discussion

### The VRN-A1 gene is body methylated and harbors additional non-CG methylation in intron 1

The 17 assays (9 bisulfite-PCR assays and 8 methylsensitive restriction-PCR assays) applied over the whole *VRN-A1* gene sequence revealed that this gene shows a non homogeneous and contrasted methylation pattern: while the 5′ and 3′ extremities are not methylated, the inside of the gene is highly methylated (Figure 2). In particular, methylation at CG sites shows a bell shape pattern with high methylation inside the gene and a low or absent methylation level at the extremities, a pattern commonly observed in plants and animals, and that is referred to as “gene body methylation” [34-37]. In *A. thaliana,* body-methylated genes tend to be longer and contain more exons than unmethylated genes [45]. Accordingly, *VRN-A1* is a long gene, with a total size of 12,233 bp and 8 exons (as compared to an average size of 3.3 kb and average exon number of 5.6 for wheat genes, [44].

In addition to the classical bell-shaped CG methylation profile, *VRN-A1* also presents a strongly methylated zone at the end of intron 1 (fragments 6.8 k(a) and 7.6 k) which involves almost complete methylation at CG sites and intermediate levels of methylation at CHG and CHH sites, a pattern that is commonly associated with TE sequences. While genes are mainly methylated at CG sites through the action of MET1 [46], DNA methylation at TEs is reinforced by the action of the RNA-directed DNA methylation (RdDM) machinery [31], which involves the production of 24 nt heterochromatic small RNAs that direct the *de novo* methylation machinery to TE sequences at CG, CHG and CHH sites. This epigenetic mechanism limits TE transposition and therefore protects the genome from insertional mutations [47-50]. Indeed the annotation of the *VRN-A1* sequence revealed the presence of several TE fragments in the second half of intron 1, *i.e*. in the region showing heavy CG, CHG and CHH methylation.

Hence, the *VRN-A1* gene is globally devoid of RdDM marks except in regions intimately associated with TE-related sequences. While this type of pattern has been observed at the genome wide level in *Arabidopsis*[51-55] the detailed gene level characterization of both *VRN-A1* methylation pattern and TE annotation makes it a good model to investigate the possible role of such intronic methylation in complex genomes.

### Vernalization hypermethylates VRN-A1 at specific CHG and CHH sites

Comparison of the DNA methylation profile of *VRN-A1* in vernalized and non-vernalized plants revealed a globally invariable pattern over most of the studied areas. A noticeable exception is the highly methylated area of intron 1, which exhibits a high level of DNA methylation in non-vernalized plants and an even higher methylation level in vernalized plants. This increase does not affect CG sites, which are almost fully methylated in non-vernalized plants and therefore likely saturated. Rather, it occurs at non-CG sites, especially CHG sites and some CHH sites (Figures 3 and 4).

The observation of a cold-induced hypermethylation at the *VRN-A1* gene raises the question of its specificity, that is, whether the hypermethylation observed is a specific response of the *VRN-A1* gene, or a consequence of a genome-wide methylation increase at non CG sites following vernalization. Environmental stress is known to induce changes in DNA methylation at the genome-wide level. In particular, a decrease in DNA methylation has been observed at the genome-wide level following vernalization in wheat [56]. Cold therefore induces a hypermethylation of *VRN-A1* which does not follow the hypomethylation trend observed at the whole genome level as reported in the study of Sherman and Talbert [56]. In this study, V1 vernalization conditions are very close to the conditions used in our study (56 days at 4°C followed by one week outside of cold room in [56], compared to 45 days at 4°C followed by 10 days in greenhouse in our study), the only difference being the tissue used (apex *vs*. leaf) and lighting (12 h light *vs*. 8 hours light). Such a cold-induced genome-wide hypomethylation has also been reported in maize [57], suggesting it is a common response in cereals. Hence, genome-wide hypomethylation also likely applies to our experiment, and points out to a specific regulation of the *VRN-A1* gene, or of a limited set of wheat genes/genomic regions.

Similarly, induction of the *Asr1* gene following drought stress in tomato has recently been associated with a change in methylation occurring within its body [58]. Although the changes observed are different (in the *Asr1* case, the stress applied induces a methylation increase at CG sites and a methylation decrease at CHH sites), these observations suggest that DNA methylation could participate in the regulation of genes involved in specific stress response. Interestingly, while in the *Asr1* case site specificity was difficult to assess due to high methylation levels in control conditions, the methylation increase observed for *VRN-A1* is clearly site-specific and restricted to the sites that are already methylated in non-vernalizing conditions. The absence of additional methylated sites suggests a reinforcement of existing methylation signal rather than the establishment of a new methylation signal.

### DNA methylation and histone modifications at the promoter

The absence of DNA methylation in the promoter of *VRN-A1* in both vernalizing and control conditions suggests that the expression change of *VRN-A1* does not occur through DNA methylation of its promoter. Both in wheat and barley, previous studies have shown that histone modification at the promoter region is involved in the transcriptional regulation of the *VRN1* gene: together with an increase in expression, vernalization treatment induces an increase in the active chromatin mark H3K4me3 and a decrease in the silent mark H3K27me3 in the barley vernalization-responsive variety Sonja [25] and increases H3K4me3 in the Norstar winter wheat cultivar [27]. Combination of these sets of information suggests that the *VRN-A1* transcriptional regulation at the promoter would be mediated by histone post-translational modifications rather than DNA methylation. Hence, even though histone post-translational modification and DNA methylation are to some extent interlinked [31,59-62], they could be disconnected in the case of the cold-response regulation of *VRN-A1*. However, distinction between the three *VRN1* homeologous copies is not clear in the Oury-Diallo study [27], thus making it difficult to assess whether the histone mark change observed is occurring at the *VRN-A1* gene promoter, at another of the *VRN1* gene promoters, or at all *VRN1* genes promoters simultaneously. For these reasons, additional studies on both histone marks and DNA methylation need to be performed for this gene on the same plant material, to test for a disconnection between DNA methylation and histone marks.

### Methylation of TE fragments in intron 1 does not follow the same trend as other TEs but likely involves the RdDM pathway

*VRN-A1* is body methylated at CG sites, but this methylation profile is not modified by cold treatment. Therefore, although *VRN-A1* is body methylated at CG, this body methylation is not associated with the expression change observed between vernalized and non vernalized plants. On the other hand, significant vernalization-induced changes in DNA methylation were found in intron 1 within a region harboring TE fragments, and occurred at CHG and CHH sites. Interestingly, TE sequences usually show a decrease in methylation and an increase in expression (and sometimes transposition) following biotic and abiotic stresses [39,63,64]. Moreover, TEs were shown to be transcriptionally activated by vernalization treatment in wheat [65]. As transcriptional activation of TEs is usually associated with a release of their silencing, our observation that the two TE fragments are hypermethylated following cold treatment suggests they are regulated by a different process.

The observation that hypermethylation occurs at cytosines from CHG and CHH contexts points out that the RNA-directed DNA Methylation (RdDM) pathway is likely involved in the hypermethylation process. Interestingly, hypermethylation is restricted to regions bearing TE fragments and targets specific CHG and CHH sites that show some methylation in non vernalizing conditions. This suggests that TE fragments could act as facilitators of DNA methylation recruitment [66]: presence of TE fragments would trigger recruitment of the RdDM machinery in control conditions, and this pathway would be even more active following cold conditions. However, because RdDM requires multiple forms of RNA polymerase activity (polymerase IV (polIV), polymerase V (polV), but also polymerase II (polII) itself [67,68]), additional investigation is required to establish whether the cold-induced methylation occurs through a higher level of PolIV/PolV transcription at TE fragments, or due to a higher PolII transcription of the gene.

While *Jorge* is a very abundant family that is estimated to harbor hundred thousands of copies in the wheat genome sharing over 90% identity with their consensus sequence (F. Choulet, pers. comm.), the fragment located on *VRN-A1* shares at most 76% identity with other *Jorge* copies (from the chromosome 3B) and is likely derived from an old insertion event. The *Sumaya* family is a middle repetitive element with less than 1000 copies in the wheat genome (F. Choulet, pers. comm.), and these copies share only 75% with their consensus, suggesting that this family did not amplify recently. The fragment of *Sumaya* LTR found in *VRN-A1* shares at most 81% identity with another *Sumaya* copy, and is therefore also an ancient insertion. Because both insertions are old and degenerated, occurrence of the RdDM pathway on these elements could be disconnected from this of the other members of these TE families.

Comparative analysis of the two TE fragments where hypermethylation occurs revealed that the *Jorge*-like transposon is conserved among the three *VRN1* copies, while the *Sumaya*-like LTR retrotransposon fragment is specific to *VRN-A1* (Figure 6). Both elements are conserved between *T. aestivum* A genome and *T. monococcum* which diverged less than 1.5 My ago [69]. This suggests they could be selectively maintained, and opens the possibility that this TE-related hypermethylation might play a role in the regulation of the gene, or in another unknown process.

### Methylation of intron 1 and gene expression

We describe the first case of a mitotically stable increase in DNA methylation in a gene intron, which is reset in the following generation. However, it remains unclear whether the changes in DNA methylation observed are a consequence of the high *VRN-A1* expression level induced by cold or if DNA methylation might play an active role in setting *VRN-A1* expression levels following cold treatment.

Several studies in wheat and barley support a functional role of promoter histone modification in setting *VRN1* expression in the triticea [25-27,70]. But deletion of *VRN-1* intron 1 is sufficient to induce a spring phenotype [28], thus suggesting a role of this intron in the regulation of *VRN-1.* Although the case of *VRN-A1* is not clear in hexaploid wheat (the only variety harboring a deletion within *VRN-A1* intron 1 also harbors a deletion of *VRN-D1* intron 1), the observation that deletion of part of *VRN-A1* intron 1 induces a spring phenotype in tetraploid wheat *Triticum turgidum* (Langdon cultivar) supports the importance of this intron for the regulation of the *VRN-A1* response to cold [28]. The recent expression comparison of *VRN-A1* expression in near-isogenic lines of wheat carrying different alleles of the gene clarified that the promoter insertion allele has a larger effect on gene expression than the intron deletion [26]. Altogether, this suggests that histone-based regulation of gene transcription occurring at the promoter is likely explaining most of the expression phenotype observed for *VRN-A1*. But this does not rule out the possibility of redundant switches in which intron 1 could participate in the regulation of the gene, with a lesser extent.

Oliver *et al*. [26] also report that the Langdon allele (Genbank accession AY747598), while still inducible by cold, has a 10 fold higher basal expression level than the wild type Triple Dirk allele (Genbank accession AY747600). Interestingly, alignment comparison of these two allele sequences reveals that deletion in the Langdon allele extends from nucleotide 1082 to nucleotide 8253 on the wild type Triple Dirk allele, thus including fragments 6.8 k and 7.6 k (data not shown). Hence, while not critical for the low-temperature response, the hypermethylated region observed could play a role in the regulation of the *VRN-A1* gene. But what role?

Recently, methylation at CHHs in the close vicinity of genes was shown to positively correlate with gene expression in maize, suggesting that this methylation mark may be interlinked with gene expression [42]. In *Arabidopsis*, DNA methylation at CG and CHG sites in the large IBM1 intron was shown to positively correlate with accumulation of the long transcriptional form of the gene, thus suggesting that intronic DNA methylation at CG and CHG sites may be required for proper IBM1-L transcript elongation [71].

DNA methylation was proposed to prevent spurious expression from cryptic intragenic promoters [36,72] or enhance accurate splicing of primary transcripts [73-75]. Interestingly, analysis of genes with long introns in *Arabidopsis* revealed that those harboring CHG methylation also tend to have TE insertions [76]. The IBM2 protein, which is enriched at intronic TEs and which mutation affects transcription of genes with long, TE-rich introns, was proposed to avoid premature transcription termination around heterochromatic domains by suppressing antisense transcription from cryptic promoters [76]. Presence of TE fragments may indeed increase the potential for cryptic transcripts, and the hypermethylation observed in *VRN-A1* intron could be involved in the taming of such transcripts. Because intron 1 is 8.5 kb long, its splicing may also be more challenging to accurately complete than the one of smaller introns. Could the inducible hypermethylation observed in TE fragments of intron 1 be associated with its splicing? In honeybee, DNA methylation in exon was shown to be positively associated with retention [77,78]. In plants, a recent study demonstrated that the novel pre-mRNA splicing factor ZOP1 is involved in both pre-mRNA splicing and the RdDM pathway in *Arabidopsis* and proposed that the splicing machinery may be involved in promoting RdDM and transcriptional silencing. A recent study indicated that inclusion of introns in transgenes can increase the transcripts levels of the transgenes in *Arabidopsis*[79], suggesting a possible role of the splicing machinery in the regulation of RNA transcript levels. Altogether, these studies suggest that the interconnection between DNA methylation, pre-mRNA splicing and transcripts levels may be more complex than previously anticipated. Further characterization of how the DNA methylation pattern observed impacts the splicing of *VRN-A1* intron 1 will likely help better understand this interconnection.

In the Langdon allele, the 7.2 kb deletion reduces intron size and deletes TE fragments, thus reducing both intron size (therefore likely facilitating splicing) and probability for cryptic promoters. Does the cold-induced hypermethylation observed reduce the impact of splicing and cryptic transcription effects?

TE fragments are remnants that are not able to move, therefore their silencing is not critical to avoid transposition. However, presence of such fragments within introns both enlarge their size (thus likely challenging splicing) and enhance chances for the emergence of cryptic promoters (thus challenging proper transcription). While additional studies are needed to further investigate the role of non CG methylation in introns, our study raises the following question: is *de novo* DNA methylation at intronic TE fragments a remaining trace of original TE silencing, or is it involved in decreasing TE-derived transcriptional effects?

## Conclusions

Our study reveals that vernalization induces hypermethylation of *VRN-A1* at specific non CG sites located in TE fragments of large intron 1, which is associated with gene expression. While additional analyses are needed to investigate whether this hypermethylation is a by-product of gene expression or participates to its regulation, even to a low extent, our study provides the first detailed DNA methylation characterization of *VRN-A1*. Observation of a cold-induced DNA methylation shift in two winter genotypes following mild vernalization treatment opens the way to examining this DNA methylation pattern in a broader set of vernalization conditions and using more time points (to get insights onto the temporal dynamics of this methylation pattern), as well as in multiple genotypes (to search for other epialleles), thus providing a starting point to investigate the biological role of DNA methylation in the wheat vernalization response. Our findings on *VRN-A1* also open interesting questions on the role of intronic CHH methylation, in particular those which are large and contain TE fragments. In particular, whether the hypermethylation observed in *VRN-A1* is also found in *VRN-1B* and *VRN-1D* copies remains to be elucidated. If a similar hypermethylation is found in all three copies, investigation of the connection of this hypermethylation to presence of other TEs in the B and D copies will help get insights into the underlying mechanism.

## Methods

### Plant material

The plant material was chosen from the wheat genetic resources built in the Dynamic Management Program headed by I. Goldringer, which have been previously characterized for *VRN1* allelic diversity [80]. In these populations, as well as in a core collection of 235 accessions [81], four polymorphic sites within the homeologous *VRN1* genes (two in *VRN-A1*, one in *VRN-B1* and one in *VRN-D1*) were found associated to earliness *per se* and vernalization requirement. At each locus, the spring form is dominant over the winter form, as well as over the other loci (epistasis), with variable degree of dominance. Among the 18 haplotypes (*i.e*., allele combination at the four sites) observed in these two studies, only three (namely *h3*, *h11* and *h14*) corresponded mostly to winter phenotypes. On the basis of genotypic and phenotypic data on earliness, two unrelated genotypes of the most frequent winter haplotype (*h3*), M91.16 and M86.04, were selected. They are referred hereafter as G1 and G2, respectively.

### Vernalization treatment

To compare the effect of vernalizing and non-vernalizing treatments, plants were sown in two sets, as presented in Figure 1. For vernalized plants, 3 biological replicates of each genotype were sown in pots in greenhouse conditions (20°C, with photoperiod of 8 hours light/16 hours dark) for ten days then transferred to a vernalization chamber (4°C, with photoperiod of 8 hours light/16 hour dark) for 45 days. After 45 days (55 days from sowing, 3 leaves stage), plants were transferred back to the greenhouse for 10 additional days, allowing the growth of the fourth leaf in non-vernalizing conditions. The control set (non-vernalized plants) was sown and kept in greenhouse conditions for 25 days. It was sown 15 days before the end of the vernalizing treatment applied to the first set, so that both sets were synchronized at the same developmental stage for leaf sampling. When fully developed, the 4th leaf of both sets was sampled and subsequently used for DNA and RNA extractions. The same protocol was repeated twice (2 sets of plants with 3 biological replicates each) except that in the second experiment, plants were grown up to the reproductive stage and vernalized plants were selfed to produce progeny, and therefore a 12 hours photoperiod was applied on all plants after vernalization. Progenies of these plants were grown until 4^th^ leaf stage in greenhouse conditions (16 hours light/8 hours night photoperiod). In this second experiment, DNA and RNA were extracted from the 4^th^ leaf and the flag leaf in parental plants and from the 4^th^ leaf in the progeny. The flag leaf was sampled 100 days after sowing (vernalized plants) and 4^th^ leaf of progeny was sampled 34 days after sowing (non vernalized plants).

### DNA and RNA extraction

DNA and RNA extractions were performed on plant tissue collected from one single leaf (4th leaf or flag leaf). DNA was extracted using DNA adsorption on Whatman Unifilter plates by following a protocol derived from the DNeasy 96 Plant kit (QIAGEN, Valencia, CA, USA). Total RNA was extracted using the TRIzol Reagent procedure (Invitrogen). Subsequent DNase treatment and DNase inactivation were carried out according to the instructions of the supplier (Ambion).

### Bisulfite analysis using direct sequencing of the converted amplified products

Sodium bisulfite treatment of genomic DNA was performed using the “EZ DNA Methylation-Gold” kit (Zymo Research), following manufacturer’s protocol. Around 350 ng of DNA were used as input for bisulfite treatment, and 2 μl of the 10 μl eluted solutions were used for each PCR reaction. Bisulfite PCR primers were designed for the plus strand, using the Methyl Primer Express v.1.0 software (Applied Biosystems). Primer design was based on the TripleDirkC sequence (Genbank accession AY747600) and is detailed in Additional file 1. Primer pairs amplifying the 9 analyzed fragments are listed in Table 1. For fragments 6.8 k and 7.6 k, which overlap with repetitive sequences, search for possible cross-amplification was performed by aligning sequences of these amplicons to available wheat shotgun sequences, using BLASTN searches (http://wheat-urgi.versailles.inra.fr/Seq-Repository/BLAST) on the IWGSC wheat shotgun sequences (http://www.wheatgenome.org/). These searches revealed that these two amplicons share only 89% nucleotide identity with the closest wheat genomic sequence found. Careful analysis of sequencing traces was also performed in order to check for presence of possible multiple peaks, which should be observed in the case of cross-amplification with repetitive sequences diverging for some SNPs. Such double-peaks were never observed, thus validating sequence-specific amplification.

PCR reactions were performed in a final volume of 50 μl containing 2 μl bisulfite-treated DNA, 1X buffer (Roche®) at 2 mM MgCl_2_, 0.25 Mm dNTP, 0.4 μM of forward and reverse primers, and 2.5 Units of hotstart Taq DNA polymerase (FastStart, Roche) using the following PCR program: an initial denaturation step (95°C, 6 min) was followed by 20 touch-down cycles (10 cycles of 94°C, 1 min; 65°C to 55°C, 1 min, 1°C decrease after each cycle; 72°C, 1 min 30 sec followed by 10 additional cycles of: 94°C, 1 min; 55°C to 50°C, 1 min, 0.5°C decrease after each cycle; 72°C, 1 min 30 sec) and 20 final cycles (94°C 45 sec; 50°C, 45 sec; 72°C, 1 min), with a final elongation step (72°C, 7 min). Size, quality and quantity of the PCR products were checked using 2% agarose gel electrophoresis. Direct Sanger sequencing of the PCR products was performed using the same primers used for PCR, without cloning. Chromatograms were analyzed for methylation quantification using the Mutation Surveyor Software (SOFTGENETICS®) with default parameters. For the validation of direct bisulfite sequencing method, two PCR products used for direct sequencing were also cloned and Sanger sequenced. Cloning was performed using the pGEM®-T vector system (Promega), following manufacturer’s protocol. Twenty clones per sample were sequenced using M13 forward and reverse primers and C/T polymorphisms among clones were visualized using the Kismeth [82].

### Restriction analysis and methyl-sensitive PCR semi-quantitative amplification

To validate the bisulfite results and to further investigate the DNA methylation pattern of regions reluctant to the bisulfite procedure, three restriction enzymes (*Bst*UI, *Msp*I and PspGI, Table 2) were used. A-genome specific primer pairs were designed to amplify 8 fragments spanning the *VRN-A1* gene sequence, each containing at least one restriction site for at least one of the above mentioned restriction enzymes (for details, see Table 3). For each enzymatic reaction, 100 ng of genomic DNA (from vernalized and non-vernalized plants) were incubated with 10 U enzyme in a final volume of 20 μl, in the following conditions: 16 hr at 37°C for *Msp*I, 2 hours at 60°C for *Bst*UI, or 1 hour at 75°C for PspGI. After each enzymatic reaction, cleavage efficiency was assessed for the different regions (primer pairs listed in Table 3) through semi-quantitative PCR, using digested DNA and genomic DNA from vernalized and non-vernalized plants as matrix. PCR reactions were performed in 30 μl with 1X Go Taq buffer, 1.5 mM MgCl_2_, 0.25 mM dNTP, 1X Q-solution (QIAGEN), 0.5 μM of forward and reverse primers, 2 μl of GoTaq polymerase (Promega) and 10 ng digested DNA using the following PCR program: initial denaturation step (94°C, 4 min) followed by 10 touch-down cycles (denaturation: 94°C, 1 min; annealing: 68°C-60°C, 1 min, 1°C decrease after each cycle; elongation: 72°C, 1.30 min) followed by 18 to 30 cycles (94°C, 45 sec; 60°C, 45 sec; 72°C, 1 min), and a final elongation step (72°C, 5 min). Five microliters of each PCR product were loaded on a 2% agarose gel and amplicon quantity was estimated using the ImageJ software [83]. Details on primer design and number of PCR cycles are described in Additional file 1.

### Gene expression analysis using qRT-PCR

30 μg of total RNA were treated with DNase (Ambion) then reverse transcribed using random hexamers (Invitrogen), 100 units of SuperScript II (Invitrogen) and 40 units of recombinant RNasin Ribonuclease Inhibitor (Promega) in a final volume of 20 μL.

A-genome specific primers were designed using Primer Express 2.0 (Applied Biosystem) with an amplicon size criterion of 150 bp and an annealing temperature of 60°C. Quantitative PCR was performed in a 25 μl total volume, with a final concentration of 300 nM of each primer. Quantitative PCR was performed using the 7500 ABI quantitative PCR system (Applied Biosystem) with Sybr-Green® as fluorophore and under the following conditions: 40 cycles of denaturation (95°C, 10 sec), annealing/elongation (60°C, 60 sec). Melting curve was made from 60 to 95°C every 0.5°C. A calibration step of the experiment was used to check for PCR efficiency. Standard curves (log of cDNA dilution vs. Ct) using serial 10-fold dilution of cDNA were built for each pair of selected primers, a 100% efficiency corresponding to a slope of -3.3 [84]. Practically, only pairs of primers yielding a slope of -3.3 ± 0.1 were selected. The specificity of the amplification (checked by dissociation curve analysis, gel electrophoresis and sequencing of the PCR product) was also assessed. From these results, one primer pair located within the 3′UTR of the *VRN-A1* gene (VRN-A1_3UTR_F: GGGCTGAGATGGCTGTACG, *VRN-A1*_3UTR_R: CAGTAGAGACGGGTATCATGG), and one primer pair located within the 18S gene (18S_F: GTGACGGGTGACGGAGAATT and 18S_R: GACACTAATGCGCCCGGTAT) were used. Although we tried several primer pairs for several housekeeping genes, only the 18S primer pair was giving good efficiency and specificity on our material. The *18S* gene being a lot more expressed than the *VRN-A1* gene in our material, a 1/50 dilution was used as matrix for the *VRN-A1* qPCRs, and a 1/100 dilution of this solution was used for the *18S* qPCRs. qPCR reactions were run by duplicate and one non-template negative control was included for each primer pair. All PCR reactions were made in the same plate. For each biological replicate, an average Ct value was calculated from the two technical replicates. Corresponding 2^ΔCt^ were then calculated for each biological replicates, and mean and standard deviation was calculated for the 4 biological replicates (2 plants from genotype G1 and 2 plants from genotype G2). Relative expression was calculated by normalizing the results by these obtained for non vernalized plants.

### TE annotation in the VRN-A1 winter genotype sequences

To check for possible presence of transposable elements within the *VRN-A1* winter allele, we ran a Repeatmasker analysis [85] with default parameters, using the *VRN-A1* sequence of the winter wheat TripleDirkC cultivar (corresponding to Genbank accession AY747600) as query and a wheat transposable elements database obtained from 18 Mb of the 3B chromosome sequence [44] as database. For each case of homology, percent identity between the TripleDirkC *VRN-A1* sequence and the sequences from the database were also extracted. Note that TripleDirkC and the two studied genotypes (G1 and G2) share the same *VRN-A1* haplotype. The same analysis was also performed on *VRN-B1* (Genbank accession AY747604) and *VRN-D1* (Genbank accession AY747606) genes from Triple DirkC, to check whether the TE described in *VRN-A1* were shared in the other two *VRN1* homeologous copies. Finally, the three *VRN1* sequences were compared using GEvo (http://genomevolution.org/CoGe/GEvo.pl) using the “BLASTN: small regions” algorithm with standard parameters. Results of this comparison and of the TE annotation were compiled to highlight which of the *VRN-A1* TEs are conserved among the three genes and which are specific to *VRN-A1*.

### Statistical analyses of methylation variation

To test the significance of methylation variation, analysis of variance was performed by using the model *Y*_*ijk*_*= μ + G*_*i*_*+ T*_*j*_*+ ϵ*_*ijk*_ where *G* represents the genotypic effect, *T* represents treatment effect (vernalizing *vs*. non-vernalizing) and *ϵ*_*ijk*_ the residual. As the *Y*_*ijk*_ are percentages, Log, Arcsin or square-root transformations of variables were performed when necessary to improve normality of residuals in the ANOVA.

## Abbreviations

FLC: *FLOWERING LOCUS C*; TE: Transposable element; PCR: Polymerase chain reaction; qPCR: quantitative PCR; RdDM: RNA-directed DNA Methylation; VRN1: *Vernalization 1.*

## Competing interests

The authors declare that they have no competing interest.

## Authors’ contributions

ARK performed bisulfite analyses, participated in methylsensitive PCR analyses and drafted part of the manuscript; JE participated in bisulfite, methylsensitive PCR and statistical analyses and coordination, and corrected the manuscript; ACM carried out methylsensitive PCRs and drafted part of the manuscript, AR carried out qRT-PCRs and performed quantitative PCR analyses, IG participated in study design and corrected the manuscript; CV participated in bisulfite and qRT-PCR analyses and coordination, performed transposable element annotations, and drafted and corrected the manuscript. JE and CV conceived and designed the study. All authors read and approved the final manuscript.

## Supplementary Material

Additional file 1 Corresponds to supplementary text. Click here for file

Additional file 2 Corresponds to supplementary figures. Click here for file
