# Supplementary material for: Vernalization treatment induces site-specific DNA hypermethylation at the VERNALIZATION-A1 (VRN-A1) locus in hexaploid winter wheat
Source: BMC Plant Biol. 2013 Dec 11;13:209. doi: 10.1186/1471-2229-13-209 (PMC3890506; doi:10.1186/1471-2229-13-209)
Supplement: Additional file 1 — Corresponds to supplementary text. [file 1471-2229-13-209-S1.pdf]

Vernalization treatment induces site-specific DNA hypermethylation at the *VERNALIZATION-A1 (VRN-A1)* locus in hexaploid winter wheat

Abdul Rehman Khan, Jérôme Enjalbert, Anne-Charlotte Marsollier, Agnès Rousselet, Isabelle Goldringer, Clémentine Vitte

Supplementary text

Comparison of direct Bisulfite Sequencing (DBS) and bisulfite-PCR-cloning

In the classical bisulfite analysis, the DNA is bisulfite treated, then PCR amplified, cloned and sequenced. Counts of C/T ratio then allows for quantifying the amount of DNA methylation at each cytosine site [1]. Here, we directly sequenced the PCR product obtained on bisulfite treated DNA, and estimated the DNA methylation level of each cytosine site by quantifying the corresponding Sanger sequence trace peaks. We refer to this technique as "direct bisulfite sequencing", or "DBS" as proposed by [2].
To validate the use of DBS to quantify DNA methylation at each cytosine site, we compared results obtained with this technique and with the classical method based on sequencing single DNA molecules after cloning the bisulfite-PCR products.

Fragment 7.6k was PCR amplified on bisulfite treated DNA from one vernalized and one non-vernalized plant and the two PCR products were directly Sanger sequenced. Methylation quantification (*i.e.*, quantification of C/T peaks corresponding to original cytosine sites) was performed on the sequence chromatogram using the Mutation surveyor® software. These two PCR products were also cloned, 20 clones were sequenced for each PCR product, and C/T ratios were estimated for each original cytosine site from sequence analysis of the 20 clones. Methylation levels detected through either software quantification or C/T counts on clone sequences were strongly correlated (R^2 = 0.958, supplementary Figure S2), thus validating the use of DBS followed by software quantification. This reliability of the DBS method has also been reported in several other DNA methylation studies, especially in human [2–4]. Note that we preferred the use of Mutation surveyor (SOFTGENETICS®) to the other available softwares (ESME, [5]; Mquant [6]) because it is not restricted to CG sites but allows for the analysis of CG, CHG and CHH sites, which are all frequently methylated in plants [7–10].

One main limit of DBS is the absence of information regarding the physical association between different methylated sites along the PCR product, while cloning and sequencing can reveal the existence of different groups of DNA molecules with contrasted DNA methylation profiles. Such contrasted profiles can be due to the mixture of different tissues or cell types during sampling. In the case of *VRN-A1*, analysis of methylation pattern between the different sequenced clones (Supplementary Figure S1) revealed a random association between methylated cytosine sites, indicating that the observed variation in the methylation state is not due to a mix of two plant tissues presenting contrasted methylation status.

Effect of primer design on Direct Bisulfite Sequencing results

Primers used for bisulfite analyses were designed to avoid presence of CG sites (only primer 11.7k F includes a CG site, for which cytosine has been replaced by the degenerate base Y; see Table 1) and to include at least 2 cytosines from non-CG context. These cytosines were considered as non-methylated and therefore converted to thymines for PCR primer design in order to favor amplification of bisulfite converted DNA templates. Primers were chosen based on *VRN-A1*, *VRN–B1* and *VRN-D1* sequence alignment to specifically target the A genome of hexaploid bread wheat. This was possible for all primers except two (primer pairs for Fragments 0.01k and 1.2k; Table 1) for which no polymorphism could be used to specifically target the A genome.

To check for the impact of presence of a C or T within the primer sequence onto the DNA methylation profile found, we developed additional primers to analyze the DNA methylation profile of fragment 6.8k(a), which contains cytosines with a wide range of methylation levels. In their 3’ half the forward primer presents two CHG sites and the forward primer harbors one CHH site. Two additional forward primers and one additional reverse primer were designed, matching Cs instead of T’s at these positions (supplementary Table S1), leading to the analysis of two new primer combinations (coded “CC/C” and “CT/C”) that were compared to the initial primer combination (coded TT/T). These three primer combinations were used to amplify fragment 6.8k(a) on bisulfite treated DNAs from one vernalized and one non-vernalized plant of the G2

genotype. These amplifications were followed by a direct bisulfite sequencing and quantification as presented in Material and Methods.

As shown in supplementary figure S3, methylation ratio estimated using the three primer combinations were identical in non-vernalized plants, while in vernalized plants the TT/T primer pair showed a slight but significant decrease as compared to CC/C and CT/C (4.8 % and 3.43 % respectively). Consequently, the C/T composition of the primers slightly affected the estimate of DNA methylation rate for fragment 6.8k but this underestimation was similar for all samples, and therefore did not affect the significance of statistical tests performed hereafter (supplementary Figure S3).

Validation of bisulfite results using methylsensitive semi quantitative PCR

For three regions showing contrasted methylation patterns with the bisulfite-based technique (0.01k, 6.8k (a) and 10.1k), we developed methylsensitive semi quantitative PCR experiments.

Primer pairs were designed to amplify fragments overlapping these three regions and containing restriction sites recognized by methyl sensitive enzymes. Details on these PCR/enzymes couples are shown in Table 3, and corresponding methylation sensitivity of the enzymes used is shown in Table 2. Example of a semi-quantitative PCR results is presented in supplementary Figure S4.

Comparison of PCR amplicon quantity obtained from genomic DNA and digested genomic DNA confirmed the results obtained with bisulfite. For fragment 0.0k, a low level of DNA methylation was verified at both CG and CHG sites. For Fragment 6.8k(b), amplicons of comparable intensity were found for both *Bst*UI-digested and non digested DNA. This result was confirmed with 17 to 24 PCR cycles, indicating that the two targeted CG sites are methylated at a level close to 100%, as observed with bisulfite. Using *Msp*I, this semi-quantitative PCR approach led to the estimation that the targeted CHG target sites is methylated at 60%. This is higher than observed using bisulfite (25% methylation for the targeted cytosine). This discrepancy, which occurs at a site with intermediate methylation level, could be due to the lack of precision of the semi-quantitative PCR method used for restriction enzyme approach, and/or to the fact that *Msp*I does not cut ^{hm5}C ^{hm5}CGG sites [11], leading to a higher amount of uncut molecules if part of the

DNA is hemimethylated (which cannot be tested using bisulfite assays designed on one DNA strand). For fragment 9.8k, a CHG site was found to be methylated at 5% using digestion but was not detected as methylated by the bisulfite treatment, a slight discrepancy that may originate from the fact that some minor peaks may stand within a limit of detection when analyzing direct sequencing traces.

Altogether, these results show that, although methylation levels vary to some extent, the results obtained with the two techniques are overall similar and not contradictory. The methylsensitive PCR approach was then used to enlarge the DNA methylation coverage of the *VRN-A1* gene by adding three additional fragments (1.6k, 3.0k, 4.0k, 5.0k and 11.0k) to our methylation analysis.

References

1. Kinoshita Y, Saze H, Kinoshita T, Miura A, Soppe WJJ, Koornneef M, Kakutani T: **Control of FWA gene silencing in Arabidopsis thaliana by SINE-related direct repeats**. *Plant J* 2006, **49**:38–45.

2. Cortese R, Lewin J, Bäckdahl L, Krispin M, Wasserkort R, Eckhardt F, Beck S: **Genome-Wide Screen for Differential DNA Methylation Associated with Neural Cell Differentiation in Mouse**. *PLoS ONE* 2011, **6**:e26002.

3. Steinfelder S, Floess S, Engelbert D, Haeringer B, Baron U, Rivino L, Steckel B, Gruetzkau A, Olek S, Geginat J, Huehn J, Hamann A: **Epigenetic Modification of the Human CCR6 Gene Is Associated with Stable CCR6 Expression in T Cells**. *Blood* 2011, **117**:2839–2846.

4. Gervin K, Hammerø M, Akselsen HE, Moe R, Nygård H, Brandt I, Gjessing HK, Harris JR, Undlien DE, Lyle R: **Extensive Variation and Low Heritability of DNA Methylation Identified in a Twin Study**. *Genome Res* 2011, **21**:1813–1821.

5. Lewin J, Schmitt AO, Adorján P, Hildmann T, Piepenbrock C: **Quantitative DNA Methylation Analysis Based on Four-Dye Trace Data from Direct Sequencing of PCR Amplificates**. *Bioinformatics* 2004, **20**:3005–3012.

6. Leakey TI, Zielinski J, Siegfried RN, Siegel ER, Fan C-Y, Cooney CA: **A simple algorithm for quantifying DNA methylation levels on multiple independent CpG sites in bisulfite genomic sequencing electropherograms**. *Nucleic Acids Res* 2008, **36**:e64.

7. Lister R, O'Malley RC, Tonti-Filippini J, Gregory BD, Berry CC, Millar AH, Ecker JR: **Highly Integrated Single-Base Resolution Maps of the Epigenome in Arabidopsis**. *Cell* 2008, **133**:523–536.

8. Lister R, Pelizzola M, Dowen RH, Hawkins RD, Hon G, Tonti-Filippini J, Nery JR, Lee L, Ye Z, Ngo Q-M, Edsall L, Antosiewicz-Bourget J, Stewart R, Ruotti V, Millar AH, Thomson JA, Ren B, Ecker JR: **Human DNA methylomes at base resolution show widespread epigenomic differences**. *Nature* 2009, **462**:315–322.

9. Cokus SJ, Feng S, Zhang X, Chen Z, Merriman B, Haudenschild CD, Pradhan S, Nelson SF, Pellegrini M, Jacobsen SE: **Shotgun bisulphite sequencing of the Arabidopsis genome reveals DNA methylation patterning**. *Nature* 2008, **452**:215–219.

10. González RM, Ricardi MM, Iusem ND: **Atypical epigenetic mark in an atypical location: cytosine methylation at asymmetric (CNN) sites within the body of a non-repetitive tomato gene**. *BMC Plant Biol* 2011, **11**:94.

11. McClelland M, Nelson M, Raschke E: **Effect of site-specific modification on restriction endonucleases and DNA modification methyltransferases**. *Nucleic Acids Res* 1994, **22**:3640–3659.

Supplementary tables

Supplementary table S1 Primer combinations designed to test the impact of possible methylated cytosine sites matching the primers. Bold letters represents cytosine positions. Modified positions are shown in italics.

Primer combination code	Primer name	Sequence (5'- 3')
TT/T (original)	6.8k(a) _F_TT	TTGTTTGTATGTGAG**T**AGA**T**TGGA
	6.8k(a) _R_T	AACTACTCCTCCACCTTAT**A**CCAA
CC/C	6.8k(a) _F_CC	GTTTGTATGTGAG***C***AGA***C***TGGA
	6.8k(a) _R_C	CTACTCCTCCACCTTAT***G***CCAA
CT/C	6.8k(a) _F_CT	GTTTGTATGTGAG***C***AGA***T***TGGA
	6.8k(a) _R_C	CTACTCCTCCACCTTAT**G**CCAA

Supplementary figures legend

Supplementary Figure S1 Lollypop graph of the clone-based DNA methylation analysis of fragment 7.6k

A. Non-vernalized plant. B. Vernalized plant. Filled or empty circles represent methylated and non-methylated cytosines, respectively. Red, blue and green colors represent CG, CHG and CHH contexts, respectively.

Supplementary Figure S2 Comparison of methylation levels quantified by DBS and bisulfite PCR-cloning-sequencing

A. Comparison for a portion of PCR fragment 6.8k(a) (vernalized plant). Top: Sanger sequence chromatogram of a PCR amplicon from bisulfite-treated DNA highlighting peaks corresponding to cytosine sites, and corresponding methylation rate estimated by the Mutation Surveyor software as percentage of C signal within each peak. Bottom: Lollypop graph representing the methylation status (given by C or T) of 20 clones obtained from the same PCR product, and corresponding C/T counts estimated over all clones. Red, blue and green colors represent CG, CHG and CHH contexts, respectively. B. Scatter plot of DBS vs. PCR-cloning-sequencing for (vernalized and non-vernalized plants were pooled for this analysis).

Supplementary Figure S3 Comparison of average methylation rates obtained on vernalized and non vernalized plants for the different primer combination designed for the PCR amplification of fragment 6.8k(a)

Grey and black represent non-vernalized and vernalized plant, respectively, from genotype G2.

Supplementary Figure S4 Example of methylsensitive semi quantitative PCR

The gel shows the results of PCR amplification of fragment 4.0k on non-vernalized G1 plant DNA digested or not with *Msp*I. Results for various numbers of PCR cycles are shown.
