# Supplementary material for: Vernalization treatment induces site-specific DNA hypermethylation at the VERNALIZATION-A1 (VRN-A1) locus in hexaploid winter wheat
Source: BMC Plant Biol. 2013 Dec 11;13:209. doi: 10.1186/1471-2229-13-209 (PMC3890506; doi:10.1186/1471-2229-13-209)
Supplement: Additional file 2 — Corresponds to supplementary figures. [file 1471-2229-13-209-S2.pdf]

A.

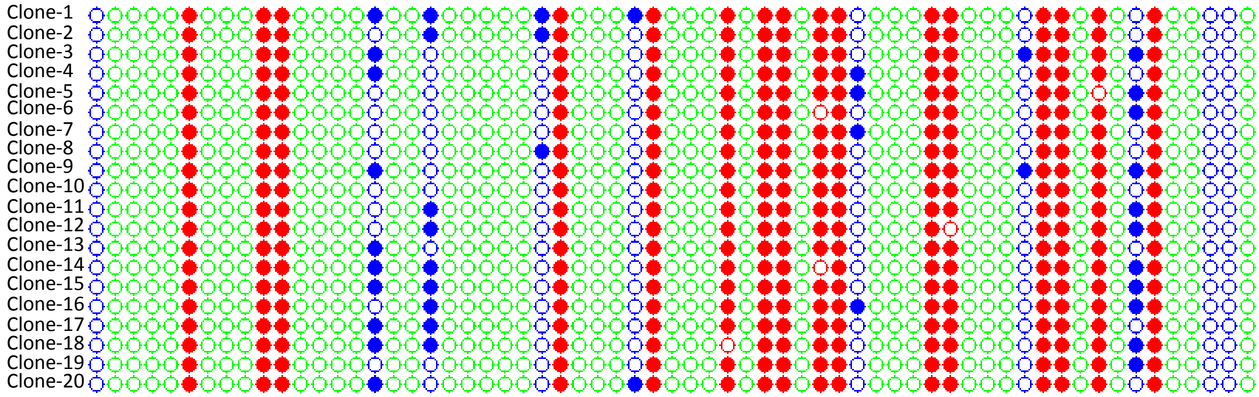

B.

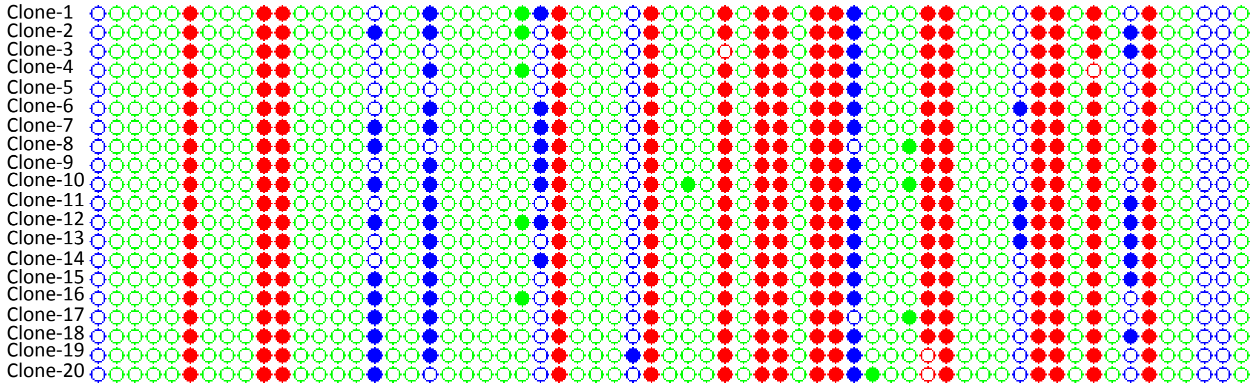

Supplementary Figure S1 Lollypop graph of the clone-based DNA methylation analysis of fragment 7.6k

A.

B.

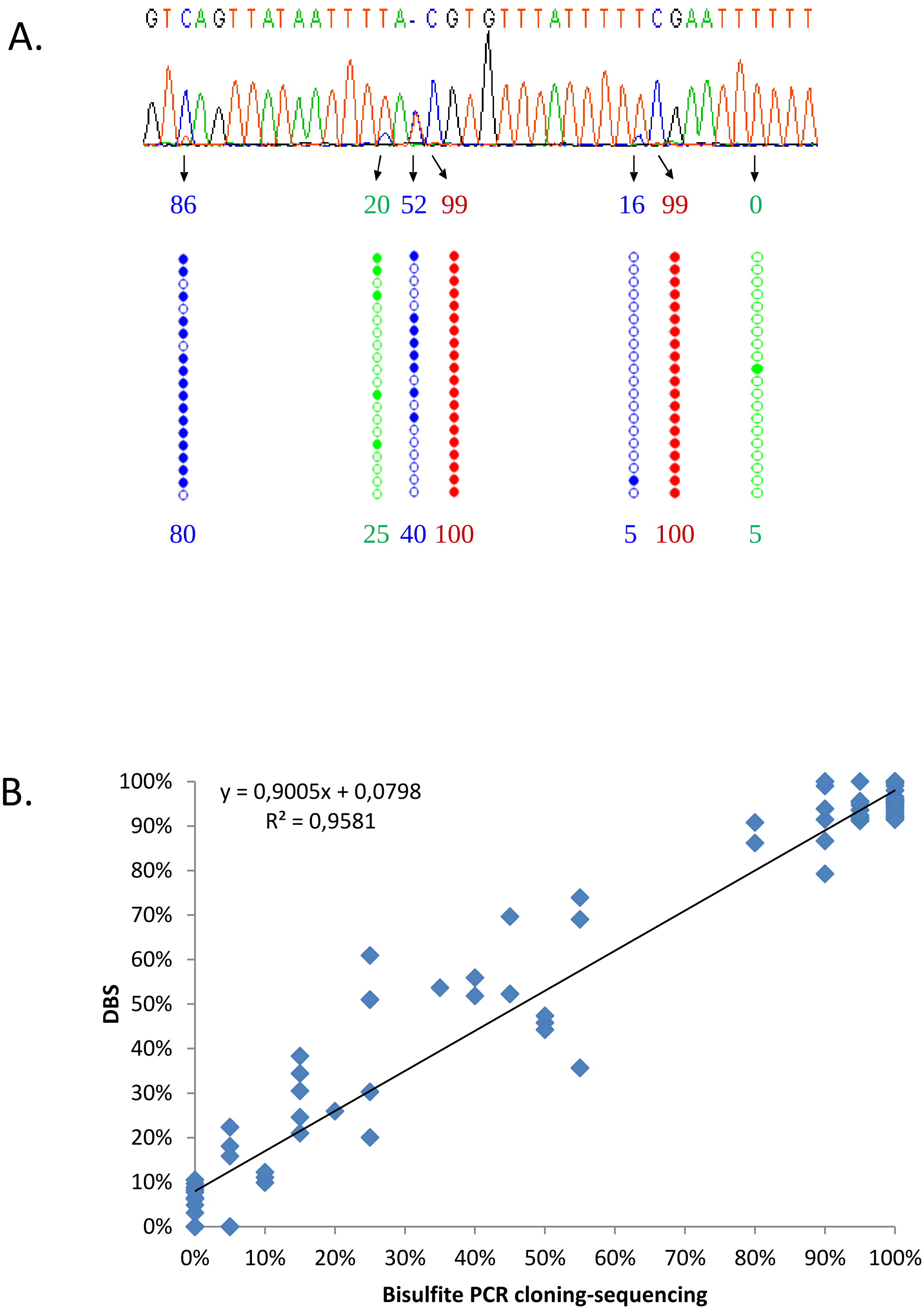

<u>Supplementary figure S2</u> Comparison of methylation levels quantified by DBS and bisulfite PCR-cloning-sequencing

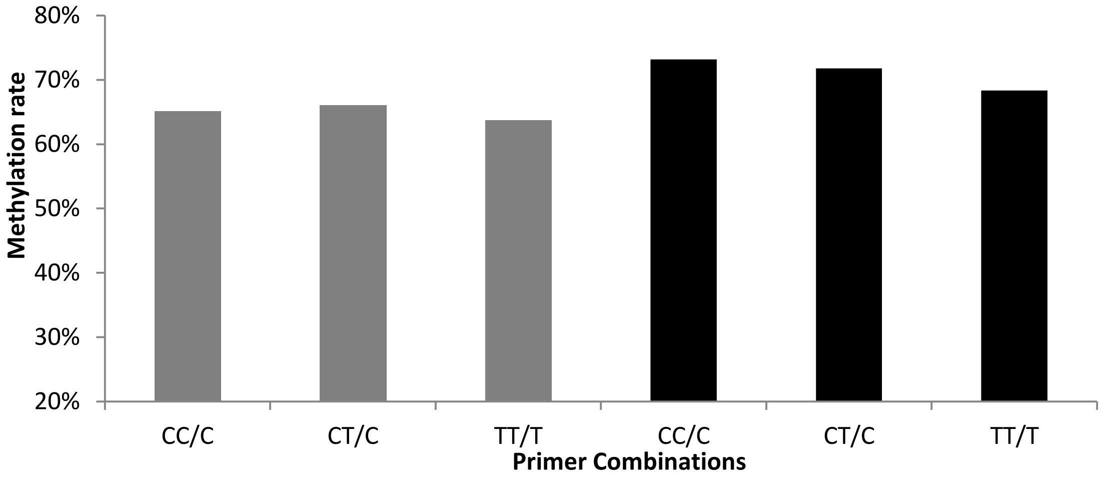

<u>Supplementary Figure S3</u> Comparison of average methylation rates obtained on vernalized and non vernalized plants for the different primer combination designed for the PCR amplification of fragment 6.8k(a)

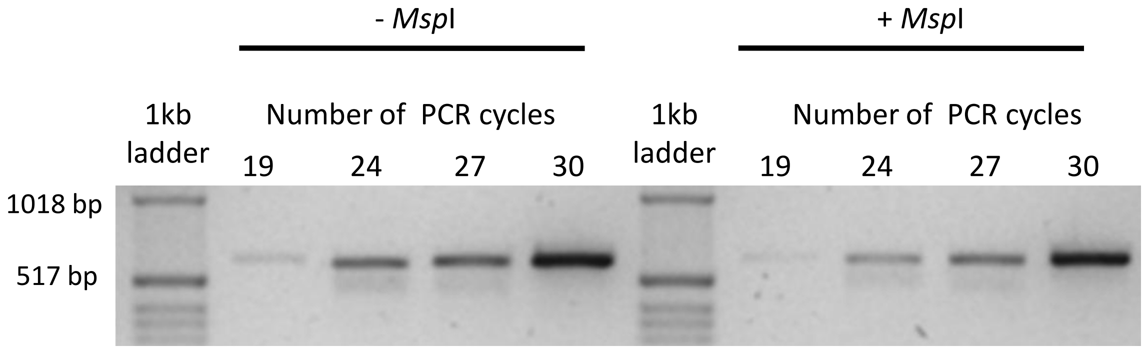

<u>Supplementary Figure S4</u> Example of methylsensitive semi quantitative PCR
